# Sensorineural hearing loss in Lyme neuroborreliosis

**DOI:** 10.1080/07853890.2024.2411014

**Published:** 2024-10-11

**Authors:** Elisa Skovgaard Jensen, Per Cayé-Thomasen, Jacob Bodilsen, Birgitte Rønde Hansen, Nichlas Hovmand, Rannvá Winther, Malene Kirchmann, Christian T. Brandt

**Affiliations:** a Department of Otorhinolaryngology, Nordsjællands Hospital, University of Copenhagen, Hillerød, Denmark; b Department of Otorhinolaryngology Head & Neck Surgery and Audiology, Rigshospitalet, University Hospital of Copenhagen, Copenhagen, Denmark; c Faculty of Health and Medical Sciences, University of Copenhagen, Copenhagen, Denmark; d Department of Infectious Diseases, Aalborg University Hospital, University of Aalborg, Denmark; e Department of Infectious Diseases, Hvidovre Hospital, University of Copenhagen, Hvidovre, Denmark; f Department of Pulmonary and Infectious Diseases, Nordsjællands Hospital, University of Copenhagen, Hillerød, Denmark; g Department of Infectious Diseases, Zealand University Hospital, University of Copenhagen, Roskilde, Denmark

**Keywords:** Hearing loss, otoacoustic emissions, Lyme neuroborreliose

## Abstract

**Background:**

Sensorineural hearing loss (SNHL) has been suggested to occur in patients with Lyme neuroborreliosis (LNB); however, a clear association has never been documented. The present study prospectively investigated the development of SNHL in patients admitted for treatment of LNB using *distortion-product-oto-acoustic emissions* (DPOAE) as a measure of cochlear function.

**Methods:**

DOAE were measured in patients with LNB on the day of diagnosis, during treatment, and 30–60 days after discharge. Frequencies were categorized as Low (1, 1.5, 2 kHz), Mid (3, 4, 5 kHz), Mid-high (6, 7, 8 kHz), and High (9, 10 kHz). Pure Tone Audiometry (PTA3) was performed at discharge and 60 days after. Patients were treated with i.v. ceftriaxone or oral doxycycline for 14 days according to guidelines.

**Results:**

DPOAE measurements were obtained in 25 patients with LNB at admission and in 18 patients at follow-up. Median age was 56 years (IQR, 48–64 years), and 16 (67%) were men. Fourteen (78%) of 18 patients showed improvement in Emission Threshold Levels (ETL) from admission to follow-up in low, mid-, and mid-high frequency categories, where ETLs increased by median levels of 3.2 (−4.1 to 8.3), 7.5 (−2.8 to 9.8), and 4.7 dB (−4.3 to 10.1). A decline was observed in the high frequency category, median −3.3 dB (−9.1 to 6.7). SNHL defined by pure tone average (PTA3) >20 dB was present in 11 out of 23 (48%) at discharge and in 9 out of 16 patients (56%) 60 days after discharge, which differed significantly from matched controls (Mann-Whitney test, *p* = 0.036).

**Conclusion:**

LNB can lead to cochlear outer-hair cell dysfunction, resulting in temporary and long-term SNHL.

## Introduction

Lyme disease is a complex infection caused by tick-borne *Borrelia burgdorferi sensu lato* spirochete. This spirochete can invade the central nervous system (CNS) resulting in Lyme neuroborreliosis (LNB) in up to 15% of the affected patients [1–3] Denmark, the incidence of LNB is 1.4–4.7 per 100.000 inhabitants per year [4, 5]. The clinical course of LNB is highly variable, but in Europe, the disease mainly manifests as subacute, painful meningoradiculitis with lymphocytic cerebrospinal fluid inflammation [2, 4–6]. LNB is a low-grade inflammatory process that, once treated, leads to a successful outcome in most patients [4, 7].

A sensorineural hearing loss (SNHL), including sudden deafness [8], has previously been suggested to be related to LNB, but with a wide range from 15 to 44% [9–11]. In addition, the hearing outcome was subject to great variation and was proposed to either sustain, fluctuate, or resolve over time [8, 12–15]. These few studies and casuistic reports are hampered by circumstantial documentation, either without comparison cohorts or without hearing assessments during and after treatment. Hearing loss was reported as a complaint in 15% of patients with long-term sequelae from LNB [9], but has not registered in more recent clinical epidemiological studies [4, 5, 7].

Since SNHL may lead to several detrimental long-term effects, including poor academic performance, poor work performance, depression, and higher risk of dementia among adults and the elderly, identification of SNHL is important.

SNHL in patients with LNB is poorly understood, and this relationship has not been systematically investigated. Therefore, the primary aim of this study was to investigate SNHL in a population with LNB from the time of diagnosis to follow-up by measuring otoacoustic emission (OAE) targeting cochlear function in patients with LNB.

## Materials and methods

### Study design

This was a prospective, observational study. Patients were included at the Department of Infectious Diseases at one university hospital from December 2017 to February 2020 (Nordsjællands Hospital, University of Copenhagen) and two university hospitals from January 2019 to June 2019 (Aalborg University Hospital and University Hospital Copenhagen Hvidovre).

### Setting and study population

Adults aged >18 years diagnosed with LNB were screened to exclude pre-existing hearing loss.

#### Inclusion criteria

Definite LNB was diagnosed based on the presence of neurological symptoms in combination with a CSF leukocyte count ≥ of 10 × 10^6^ cells/L and positive intrathecal *B. burgdorferi sensu lato* antibody production. Probable LNB was diagnosed based on the presence of neurological symptoms suggestive of Lyme neuroborreliosis in combination with a CSF leukocyte count ≥ of 10 × 10^6^ cells/L and a positive blood *B. burgdorferi sensu lato* antibody test, and no other cause of CNS inflammation was identified [5, 16].

#### Exclusion criteria

Pre-existing hearing loss, head trauma, significant history of noise exposure, ear surgery, concurrent or previous treatment with ototoxic agents (e.g. gentamycin), prior central nervous system pathology, and a history of prior central nervous system infections, including LNB.

### Serology

#### Borrelia-specific antibodies in serum

For the detection of serum-*B. burgdorferi sensu lato* antibodies, IDEIA *B. burgdorferi sensu lato* IgG and IgM (Oxoid Hampshire, UK) were used.

#### Borrelia-specific intrathecal antibody index (AI)

The IDEIA flagella antigen-based enzyme-linked immunosorbent assay LNB test (Oxoid Hampshire, UK) was used to detect intrathecal synthesis of *B. burgdorferi sensu lato* specific IgG and IgM antibodies. An AI > 0.3 was considered positive according to the manufacturer’s instructions [5].

## Hearing assessment: pure tone audiometry

The patients were tested upon discharge and 30–60 days after discharge. Patients underwent pure-tone audiometry at frequencies of 0.125–8 kHz. Both air and bone conduction were measured using (*Madsen^®^ Astera 2 Clinical Audiometer*). The pure-tone average (PTA) was calculated as the average of the thresholds at 0.5, 1, 2 and 4 kHz (PTA3). In cases of conductive hearing loss, bone conduction was performed. Each ear was classified as follows: no hearing loss (≤ 20 dB HL), mild (21–40 dB HL), moderate (41–55 dB HL), moderately severe (56–70 dB HL), severe (71–90 dB HL), or profound (> 90 dB HL) (24). The PTA of each patient was compared to PTA from an age- and sex-matched normative dataset provided by ISO-7029 [17].

### Distortion product otoacoustic emissions (DPOAE - OAE)

Otoacoustic emissions (OAE) were recorded using an Interacoustics Titan DPOAE 440 module. Eleven frequencies were measured in each ear: 1 kHz, 1.5, 2, 3, 4, 5, 6, 7, 8, 9 and 10 kHz. The frequency ratio (f2/f1) was fixed at 1.22. OAE was measured bedside with patients lying down on their backs with a 30°head position. OAE thresholds were registered for each ear. Otoscopy and tympanometry preceded each measurement; in cases of outer or middle ear pathology, the affected ear was excluded. The cerumen that might have interfered with the measurements was removed.

Frequencies were categorized as low (1, 1.5, 2 kHz), mid (3, 4, 5 kHz), mid-high (6, 7, 8 kHz) and high (9, 10 kHz). The emission threshold level (ETL) of the otoacoustic emissions is presented as the mean ETL in decibels (dB) of the frequencies included in each category. There were no significant differences in ETLs between the right and left ears. Therefore, the mean ETL of both ears was used to determine ETLs [18, 19].

OAE thresholds were obtained in three intervals:

1. Admission: Within 24 h from diagnostic lumbar puncture and initiation of treatment.
2. Treatment: During treatment days 1 to 3 and 5 to 7.
3. Follow-up in the outpatient clinic 30 to 60 days after discharge.

#### Noisefloor

A signal-to-noise ratio (SNR) of +3 dB was applied to the noise floor at low to mid-high frequencies and +6 dB at high frequencies, as previously described [18, 20]. The distribution of the final noise floor, and thus the border of the OAE detection, within each frequency category was: low −10 dB, mid −15 dB, mid-high −13 dB, high −16 dB.

##### OAE healthy control group

**Figure 1.**
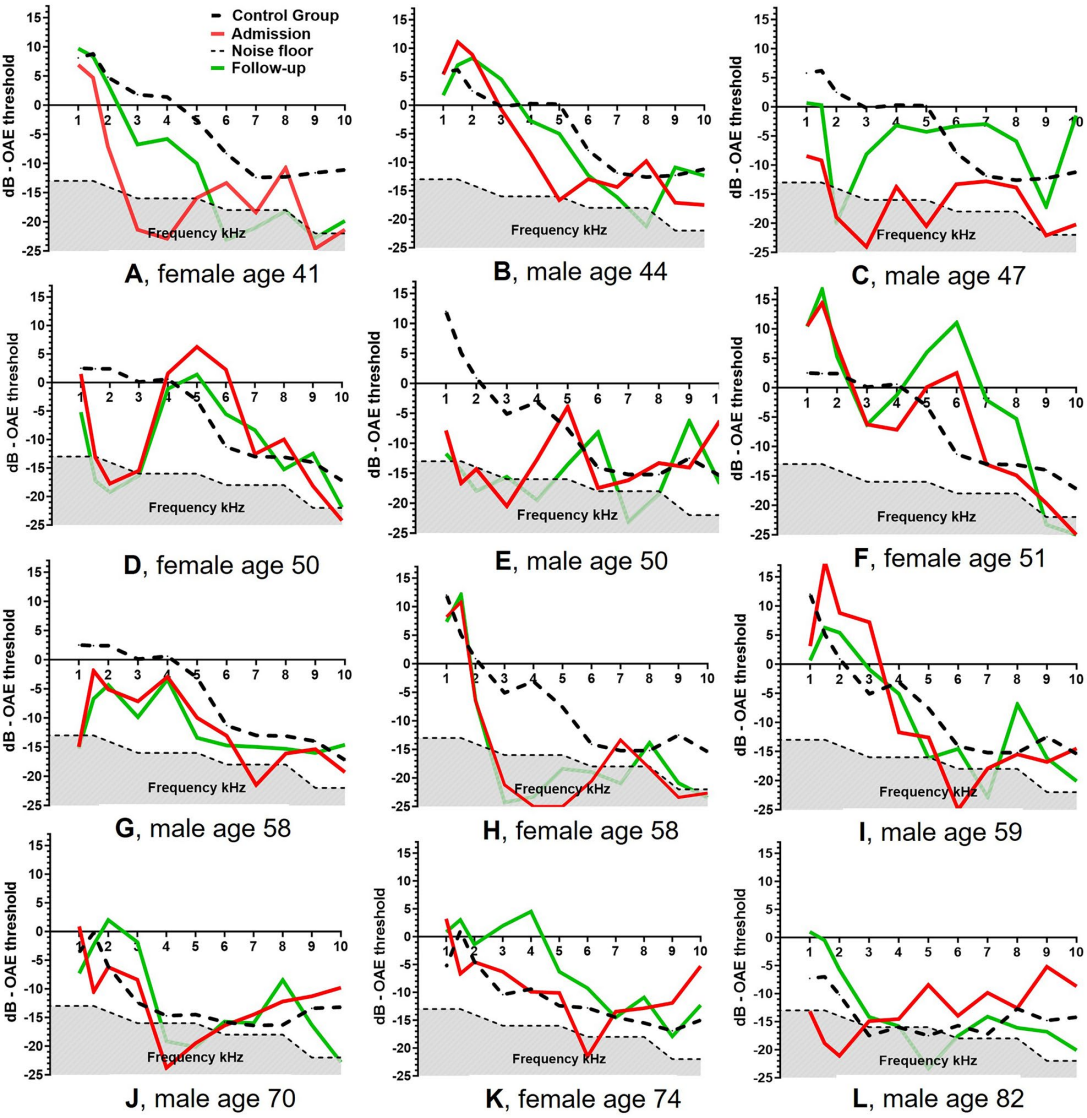
Emission levels in dB—OAE threshold—in 11 frequencies (1, 1.5, 2, 3, 4, 5, 6, 7, 8, 9 and 10 kHz). DPOAE on admission (RED line) and follow-up (GREEN line) in 12 patients with Lyme neuroborreliosis. The thick dotted line shows the mean emission levels in an age- and sex matched control group. The dotted thin line with dark grey area below delineates the noisefloor. Patient A and C complained of hearing loss during admission. Patient L, a male aged 82 complained of hearing loss at follow up.

Distortion product otoacoustic emissions (DPOAE) were obtained in 158 healthy persons with an even sex distribution divided into decennials from 18 to 80+, as previously described [19]. Data are presented in Figure 1, showing the mean emission threshold levels among age- and sex-matched healthy adults.

### Statistical evaluation and data handling

Data analysis and graphical presentations were performed using GraphPad PRISM v. 9.0 and R studio version 1.4.1717. Fisher’s exact test and Mann-Whitney U test were used to compare groups, and Spearman’s rank test was applied for the correlation analysis between PTA and ETL levels. Statistical significance was set at *p* < 0.05.

Changes in ETL from admission to final follow-up were calculated as the difference between the patient’s own ETL on admission and follow-up (mean of both ears). A recovery or decline of ≥2.5 dB was considered significant, and changes below 2.5 dB were registered as “0” (zero) [18, 20]. As described above, the noise floor + SNR was used as the lowest detectable emission. Emission changes in the four frequency categories were pooled to compare changes in ETLs during admission and from admission to follow-up, emission changes in the four frequency categories were pooled for analysis.

### Ethics

This study was approved by the Danish Data Protection Agency (2012-58-0004, I-Suite 03637). Written informed consent was obtained from the patients before enrolment. The Regional Committee on Health Research Ethics in the Capital Region did not require registration, as the study did not interfere with patient treatment, and testing with OAE was harmless (protocol no. H-1-2012-086, author CTB). The study adheres to the Declaration of Helsinki. This study was registered at ClinicalTrials.gov (identifier: NCT03715569).

## Results

In total, 29 patients with LNB were screened for inclusion. Four patients were excluded because of pre-existing hearing loss. One patient only had an OAE obtained on admission. Eighteen of 25 (72%) patients had definite LNB and 7 of 25 (28%) probable LNB (serology in Supplemental table 1). The median age was 56 years (IQR, 48–64 years), and 16 (67%) were men.

The clinical presentation and laboratory results are presented in Tables 1 and 2, respectively. None of the patients received treatment for LNB before admission or diagnosis.

**Table 1.** Clinical characteristics in 25 patients with Lyme neuroborreliosis.

Clinical characteristics	No. of patients
Age, years	56 [48–64]
Sex, male	16 (67%)
History of tick bite	12 (50%)
History of erythema migrans	7 (29%)
Temperature on admission, Celsius	36.9 [36.7–37.4]
Total duration of antibiotics treatment, days	14 [14–14]
Duration of symptoms before admission, days	14 [14–21]
Facial nerve paresis	10 (40%)
Abducens nerve paresis	1 (4%)
Radicular pain	8 (32%)
Peripheral nerve palsy	7 (28%)
Dizziness	2 (8%)
Fatigue	2 (8%)
Memory impairment	4 (16%)
Bladder paresis	2 (8%)

Data shown as medians with interquartile range. Numbers with percentage in parenthesis.

**Table 2.** Laboratory results of 25 patients with Lyme neuroborreliosis.

Laboratory results	Included patients (*n* = 25)	Reference interval
P-CRP (mg/L)	3 [3–5]	<10
B-leukocytes (10^9^/L)	7.4 [5.6–10.1]	3.5–8.8
B-thrombocytes (10^9^/L)	225 [196–286]	145–390
P-creatinine (µmol/L)	67 [58–75]	50–100
CSF Leukocytes (10^6^/L)	143 [44–294]	<5
CSF mononuclear cells (10^6^/L)	134 [43–290]	<5
CSF glucose (mmol/L)	3.3 [2.7–3.7]	2.2–3.9
CSF protein (g/L)	1.6 [0.9–2.3]	0.15–0.50
CSF lactate (mmol/L)	2.6 [2.1–3.4]	1.1–2.4

Data shown as medians with interquartile range.

**Table 3.** Loss and recovery of emissions from admission to follow up.

	Study interval **-** days	No. with emission change in one or more frequency areas		Change in emission threshold levels (ETL) in decibel - dB			
			n/n	Low frequency 1, 1.5, 2 kHz	Mid frequency 3, 4, 5 kHz	Mid-high frequency 6, 7, 8 kHz	High frequency 9, 10 kHz
All patients	1 to 3	_	6/11	2.5 (−4.4 to 2.6) *n* = 3	−5.0 (−6.2 to 2.7) *n* = 4	−4.1 *n* = 1	2.8 and 3.4 *n* = 2
	1 to 7	_	7/11	−7.2 and 6.9 *n* = 2	−5.1 and 2.6 *n* = 2	−4.9 (−7.8 to 5.3) *n* = 5	−6.3 and 5.5 *n* = 2
	1 to >30	_	14/18	3.2 (−4.1 to 8.3) *n* = 8	7.5 (−2.8 to 9.8) *n* = 5	4.7 (−4.3 to 10.1) *n* = 7	−3.3 (−9.1 to 6.7) *n* = 6
	14 to >30	_	3/3	3.3 *n* = 1	−2.7 (−3.3 to 8.9) *n* = 3	−3.3 *n* = 1	−6.5 *n* = 1
Gender Female Male	1 to >30	F	6/7	−4.1 and 5.7 *n* = 2	4.8 (−2.8 and 7.5) *n* = 3	5.5 (−2.7 to 10.1) *n* = 4	−3.2 *n* = 1
		M	8/11	3.2 (−2.8 to 8.3) *n* = 6	7.5 and 9.8 *n* = 2	−1.4 (−4.3 to 8.9) *n* = 4	−3.3 (−9.1 to 6.7) *n* = 5
Definate or Probable LNB	1 to >30	D	9/13	3.6 (−4.1 to 8.3) *n* = 6	6.9 (−2.8 and 9.8) *n* = 4	5.5 (−4.3 to 10.1) *n* = 6	−4.9 (−9.1 to 6.7) *n* = 4
		P	5/5	2.8 (−2.8 to 5.7) *n* = 3	7.5 and 7.5 *n* = 2	−2.8 *n* = 1	−3.2 (−4.4 to −3.7) *n* = 3
Diagnostic delay - Days	1 to >30	0 − 14	7/10	−0.5 (−2.8 to 5.7) *n* = 4	7.5 (7.5 to 9.8) *n* = 3	−2.8 (−4.3 to 8.9) *n* = 3	−3.3 (−4.4 to 6.7) *n* = 4
		>14	7/8	3.2 (−4.1 to 8.3) *n* = 4	−2.8 and 4.8 *n* = 2	5.5 (−2.7 to 10.1) *n* = 4	−9.1 and −2.7 *n* = 2

Data shown as medians with full range (min/max) in each frequency category. The number of patients with a ETL change in each frequency category is registered below the ETL change in dB. If fewer than 3 patients had changes in ETL the actual ETL change in dB and not median is provided. The emission change in dB was calculated as the difference in each patient’s ETL in the designated time interval (days 1 to 3, days 1 to 7, or days 1 to 30). The lowest level of emission detection was the noise floor where an SNR of +3 dB (low-, mid and mid-high frequencies) and +6 dB (high frequencies) was applied.

### Treatment

All patients were treated with antibiotics for 14 days according to the Danish national guidelines. Seventeen (68%) patients were treated with iv ceftriaxone 2 g daily upon admission; twelve of these patients were 4 to 5 days later switched to oral doxycycline. Eight (32%) patients were treated with oral doxycycline throughout the whole treatment period.

### Distortion Product OtoAcoustic Emissions (DPOAE) (Figure 1 and Table 3)

Twenty-two (88%) patients had their first OAE performed on admission, of which 18 (72%) patients were available for comparison of ETLs from admission to follow up. Three (13%) patients had an OAE obtained after end of treatment and at follow-up of >30 days. In 11 (46%) patients, OAE was available during admission until day 7.

Two patients had spontaneous complaints of hearing loss on admission (Figure 1A, C), and one patient complained of hearing loss at follow-up (Figure 1L).

### *OAE during admission (*Table 3)

Between days 1 and 3, a significant change in ETLs was found in 6 of 11 patients (55%) and in 7 (63%) of 11 patients until day 7. Four patients showed no changes in the ETL during this period. The no. of frequency categories with a decline in ETL was higher on day 7 than in frequency categories with recovery (7 vs. 3).

### OAE from admission to follow-up (Table 3)

Fourteen (78%) of 18 patients showed significant changes in ETLs from admission to follow-up. In low, mid- and mid-high frequency categories, ETLs increased by median levels of 3.2 (−4.1 to 8.3), 7.5 (−2.8 to 9.8) and 4.7 dB (−4.3 to 10.1), respectively, whereas a net decline was observed in the high frequency category, median −3.3 dB (−9.1 to 6.7).

### Gender and changes in ETL (Table 3)

No differences were found in ETL changes from admission to follow-up between female patients (*n* = 7) and male patients (*n* = 11) (Mann-Whitney *U*-test, *p* = 0.62).

Including patients examined from the end of treatment (day 14) and at least 30 days after treatment (female = 1 and male = 2) did not change the results (Mann-Whitney *U*-test, *p* = 0.3).

### Definite or probable LNB (Table 3)

No differences were found in ETL changes from admission to follow-up in patients with definite LNB (*n* = 13) compared with patients with probable LNB (*n* = 5) (Mann-Whitney *U*-test, *p* = 0.61). Including patients examined from the end of treatment (day 14) and at least 30 days after (definite LNB, *n* = 3) did not change the result (Mann-Whitney *U*-test, *p* = 0.74).

### Duration of symptoms (Table 3)

Median duration of symptoms before diagnosis was 14 days (IQR, 14–21 days). Comparing ETL changes in patients with a symptom duration of up to 14 days to ETL changes in patients with symptoms for more than 14 days no differences were found (Mann-Whitney *U*-test, *p* = 0.74). Including patients examined from the end of treatment (day 14) and at least 30 days after treatment (≤14 days, *n* = 2 and >14 days, *n* = 1) did not change the result (Mann-Whitney *U*-test, *p* = 0.38).

### Clinical presentation: symptoms and focal neurology

The most common neurological signs or symptoms were facial nerve palsy (*n* = 10, 40%), radicular pain (*n* = 8, 32%), and other peripheral nerve palsies (*n* = 7, 28%), see Table 1). No differences in ETL were found between the facial paralytic and non-paralytic sides on admission (Mann-Whitney, *p* = 0.48) or at the final follow-up (Mann-Whitney, *p* = 0.99).

### Pure-tone-audiometry (Table 4)

Pure-tone audiometry was performed at discharge (first audiometry) and follow-up (second audiometry). In total, 23 of 25 patients underwent the first audiometry (92%), and 16 patients (64%) underwent the second audiometry, see also Table 4. The seven patients that did not show up for their second audiometry had no hearing loss (*n* = 4), mild hearing loss (*n* = 2), or moderate hearing loss (*n* = 1) in the first audiometry.

**Table 4.** Classification of HL in patients with LNB with and without age-and-sex adjustment.

Severity of hearing loss	First audiometry	Second audiometry
No	12 (52%)	7 (44%)
Mild	9 (39%)	8 (50%)
Moderate	2 (9%)	1 (6%)
Severe	0 (0%)	0 (0%)
Profound	0 (0%)	0 (0%)

Classification is based on PTA in the worst ear. No hearing loss (≤ 20 dB HL), mild hearing loss (21–40 dB HL), moderate hearing loss (41–55 dB HL), moderate-severe (56–70 dB HL), severe (71–90 dB HL), profound (> 90 dB HL). Audiometry was performed 5 days (median) and 57 days (median) after discharge. First audiometry (*n* = 23), second audiometry (*n* = 16).

Hearing loss, defined by worst ear, was present in 11 (48%) in the first audiometry and 9 (56%) in the second audiometry. SNHL was bilateral in all cases except in one patient.

Among the 9 patients with hearing loss in the second audiometry, mild hearing loss remained unchanged from the first audiometry in 6 patients. One patient had late-onset mild hearing loss, and in one patient, hearing improved from moderate to mild hearing loss. Furthermore, in one patient, hearing declined from mild hearing loss during the first audiometry to moderate hearing loss. Hearing loss was pronounced at the highest frequencies (4–8 kHz). PTA from the first audiometry was significantly lower than that from the age-and sex-matched control dataset (Mann-Whitney test, *p* = 0.036).

### DPOAE and PTA

The ETL average at the final follow-up in frequencies 1–4 kHz was significantly correlated with the PTA results of the first audiometry (*p* = 0.0007, Spearman’s rank −0.71).

## Discussion

The present study is the first to measure cochlear function over time using highly sensitive otoacoustic emission (OAE) in patients with LNB. In addition, this is likely the first firm documentation of cochlear involvement in LNB, and thus the occurrence of temporary and permanent hearing loss in LNB [11]. We found that cochlear function was temporarily or permanently reduced in two-thirds of the patients. ETLs at frequencies from 3 to 8 kHz were subject to a decrease during the first week of admission, although fluctuations over days in ETLs across all frequency groups were present during treatment. From admission to the final follow-up, one-third of the patients showed recovery of cochlear function, equivalent to improved hearing. This was most pronounced at mid- and mid-high frequencies, where ETLs increased up to 10 dB. A decline in ETLs upon follow-up was detected in the high-frequency category, equivalent to high-frequency hearing loss from LNB.

Although LNB differs from bacterial meningitis in terms of timely evolution, clinical presentation, and CSF biochemistry, our clinical findings do agree with the cochlear pathology described in bacterial meningitis, in which inflammation is pronounced in the basal turns of the cochlea, resulting in high-frequency hearing loss [21]. This can, to some extent, explain the better recovery of lower frequencies because they are situated further away from dense infiltrates in the basal cochlea. However, we expected that the group of patients with the longest diagnostic delay would have a poorer recovery, which was not observed in our population.

The fluctuation in ETLs observed in the early treatment period of LNB may reflect early effects of treatment but can also be part of the nature of LNB with a slow progression, thus leading to an initial decline in ETLs. However, this is speculative.

To the best of our knowledge, cochlear function in LNB has only been assessed by OAE once before [14], in which a pediatric patient with a symptom duration of 5 months presented with bilateral SNHL, almost completely absent OAE on both ears, and a bilateral normal auditory brain stem response (ABR). Follow-up examination 1.5 years later showed near-normal PTA and full emission recovery in one ear. This finding agrees with our results, indicating that the cochlea is a site of dysfunction and even injury.

Cochleitis was detected on MRI with inflammatory changes in the basal and middle turns of the cochlea in an LNB case report [22], although MRI findings are not necessarily related to SNHL [23]. Cochlear inflammation *via* the spiral ligament capillary bed, as well as oxidative stress causing outer hair cell apoptosis, may be other possible explanations for cochlear dysfunction. We propose that the mechanism leading to cochlear dysfunction and SNHL in LNB is similar to the pathogenesis of cochlear dysfunction in bacterial meningitis, although the spirochaete is unlikely to be neurotoxic, and the inflammatory process is much less pronounced.

Borrelia meningoradiculitis (BM) is the most common manifestation of LNB in Europe. Symptoms may include headache, cranial nerve palsy, and lancinating radicular pain, and which up to 80% of European patients present with facial nerve palsy [5]. The relationship between facial nerve paralysis and hearing loss is unclear; however, we found no association between the site of facial nerve paralysis, which was mainly unilateral, and bilateral hearing loss.

The study cohort matched earlier Danish cohorts [4] in terms of symptoms, age, sex, biochemistry, and time from symptom onset to treatment; therefore, even though our cohort was relatively small, we believe that our cohort is representative, and our findings are generalizable.

### Limitations

Our sample size was relatively small and did not include patients diagnosed and treated in outpatient clinics. This likely skewed our study to include patients with more advanced/disseminated disease. In addition, the limited sample size did not encourage further analysis of factors associated with emission loss.

Furthermore, the clinical impact of ETL changes is likely subclinical in many cases. However, our method, in which 11 individual frequencies are grouped into four main frequency categories, may obscure changes in single frequencies. Lastly, patients may have had pre-existing undisclosed hearing loss despite our screening.

### Future perspectives

To preserve outer hair cell function, limiting diagnostic delay is important. However, this requires further investigation. Adjunctive dexamethasone and antioxidant therapy have been proposed to be useful in treating bacterial meningitis and may also be a future treatment option for LNB. Although SNHL was common in our group of patients with LNB, new studies are needed to make recommendations regarding systematic audiological screening in patients with LNB, other than those with specific complaints. Our results suggest that DPOAE could be included as an objective measure in future studies of LNB treatment and factors related to long term neurological sequelae.

## Conclusion

LNB can cause outer hair cell dysfunction in the cochlea, resulting in temporary and long-term SNHL. Early diagnosis and treatment are essential to reduce the risk of cochlear injury.

## Supplementary Material

Supplemental Material.docx

## Data Availability

Due to strict data-sharing policies in Denmark, we are unable to make our data publicly available. However, researchers who wish to access the data can do so by contacting Associate Professor Christian Brandt (chtb@regionsjaelland.dk) at the Department of Infectious Diseases, Zealand University Hospital or the Regional Data Protection Agency (forskningfortegnelse@regionsjaelland.dk) to create a data-sharing contract. After permission has been granted by the regional data committee, the data will be made available to the researchers.
