# Supplementary material for: Sensorineural hearing loss in Lyme neuroborreliosis
Source: Ann Med. 2024 Oct 11;56(1):2411014. doi: 10.1080/07853890.2024.2411014 (PMC11486336; doi:10.1080/07853890.2024.2411014)
Supplement: Supplemental Material.docx [file IANN_A_2411014_SM2404.docx]

**Supplemental Table S1**. The presence of serum antibodies to B. burgdorferi sensu lato and cerebrospinal fluid antibody index (AI) findings in 25 patients with Lyme neuroborreliosis.

|  | Serum | | | | |
| --- | --- | --- | --- | --- | --- |
| AI findings | Positive IgM | Positive IgG | Positive IgM+IgG | Negative IgM+IgG | Total No. of patients |
| Positive AI IgM | 1 | 2 | 1 | 0 | 4 |
| Positive AI IgG | 0 | 0 | 0 | 0 | 0 |
| Positive AI IgM+IgG | 0 | 8 | 6 | 0 | 14 |
| Negative AI IgM+IgG | 0 | 4 | 3 | 0 | 7 |
| Total No. of patients | 1 | 14 | 10 | 0 | 25 |
